# Movement in High School: Proportion of Chinese Adolescents Meeting 24-Hour Movement Guidelines

**DOI:** 10.3390/ijerph17072395

**Published:** 2020-04-01

**Authors:** Li Ying, Xihe Zhu, Justin Haegele, Yang Wen

**Affiliations:** 1 School of Sport and Physical Education, Huainan Normal University, Huainan 232038, Anhui Prov, China; yl0608@hnnu.edu.cn; 2 Department of Human Movement Sciences, Old Dominion University, Norfolk, VA 23508, USA; x2zhu@odu.edu (X.Z.); jhaegele@odu.edu (J.H.); 3 Center of Jiangsu Sports Health Engineering Collaborative Innovation, Nanjing Sport Institute, Nanjing 210014, Jiangsu Prov, China

**Keywords:** adolescence, gender, screen time, sleep, physical activity, prevalence

## Abstract

The purposes of this study were (a) to examine the proportions of adolescents in China who partially or fully meet three 24-h movement guidelines on physical activity, screen-time, and sleep duration and (b) to examine whether there were gender differences in the proportion of boys and girls meeting these guidelines. The sample was made up of high school adolescents from an eastern province of China (*N* = 1338). The participants completed a self-reported survey on demographic variables and weekly health behaviors including physical activity, screen-time, and sleep duration. A frequency analysis was conducted to summarize the number of 24-h movement guidelines met of the total sample and by gender; chi-squared tests were used to examine the gender differences in the proportion of students meeting different guidelines, independently and jointly. A high proportion of adolescents did not meet physical activity (97.2%, 95% CI = 96.2–98.0%), or sleep (92.1%, 95% CI = 90.6–93.5%) guidelines, but met screen-time (93.6%, 95% CI = 92.4–94.7%) guidelines. Overall, only 0.3% (95%CI = 0.1–0.6%) of the sample met all three guidelines, 8.8% (95%CI = 7.5–10.2%) met two, 85.8%% (95%CI = 84.0–87.4%) met one, and 5.1% (95%CI = 4.0–6.4%) met none. There was no statistically significant percentage difference between female and male participants in meeting physical activity, screen-time viewing, or sleep duration guidelines, independently or jointly (*p* values > 0.05). These figures of participants meeting all three guidelines or physical activity and sleep independently are much lower than many estimates in prior research internationally. Considerations to improve adherence to physical activity and sleep guidelines are critical in this population.

## 1. Introduction

Pediatric obesity is often cited as a major and pressing health concern [1,2] that can lead to numerous physiological (e.g., high blood pressure, type 2 diabetes) and psychological (e.g., depression, poor quality of life, low self-esteem) issues throughout the lifespan [3,4,5]. Consistent with international trends [1], national data has demonstrated that China has experienced an alarming increase in pediatric obesity in the past 20 years, as well as associated physiological conditions (i.e., hypertension, diabetes) [6,7]. According to Wang and colleagues [6], this steady increase in obesity in China has coincided with the country’s rapid economic growth, and may be a result of multiple lifestyle factors, including unhealthy diet, lack of physical activity (PA), and increased sedentary behavior. With this increase in pediatric obesity, estimates in 2013 suggested that approximately 23% of boys and 14% of girls in China were overweight or obese [1], which is comparable to many Western countries. Of further concern, China is projected to have the highest number of obese children in the world by 2030 [8,9].

Given the deleterious effects associated with pediatric obesity, close monitoring of children’s weight and the promotion of healthy lifestyle behaviors are of concern in China [6,10]. PA, sedentary behavior, and sleep duration are codependent lifestyle behaviors that together account for the 24-h daily cycle [11,12] and may be of interest with regard to reducing pediatric obesity [13,14,15,16,17,18]. Reflecting the belief that the whole day matters, recent years have seen the development and adoption of movement-related recommendations across the 24-h period [11,12,13]. For example, the 24-h movement framework presented by Tremblay and colleagues [11] includes recommendations for adolescents aged 14–17 years to engage in (a) at least 60 min of moderate-to-vigorous PA, (b) two or less hours of recreational screen time, and (c) 8 to 10 h of sleep per night. Central to the integrated nature of the 24-h movement framework paradigm is the concept that meeting all three movement behavior recommendations may have a greater association with beneficial health outcomes, such as reducing the risk of pediatric obesity, than meeting a single guideline [12]. Supporting this assertion, an emerging body of evidence suggests that meeting all three 24-h movement guidelines is associated with lower rates of overweight and obesity [13,14,15,16,17,18], as well as other favorable physiological [16,17] and psychological outcomes [5] during childhood and adolescence. 

The emergence of this more holistic and comprehensive perspective of health behaviors, which supports the integrated benefits of meeting all three 24-h movement guidelines, has prompted research in a number of countries that examines the proportions of youth who meet these guidelines [13,19]. For example, a recent cross-sectional study [12] using nationally represented data from the 2016–2017 National Survey of Children’s Health in the US demonstrated that just 9.4% of youth aged 10–17 years met all three 24-h movement guidelines, whereas nearly the same amount (9.1%) met none of these guidelines. Similarly, compliance with 24-h movement guidelines was recently reported to be alarmingly low among adolescents in Hong Kong, where just 1.0% of a sample of 692 adolescents (aged 11–17 years) met all three guidelines. The current body of evidence suggests that just a small percentage of youth in these studies are experiencing the benefits associated with meeting each of the three 24-h movement guidelines [13,14,15,16,17,18]. 

Given issues concerning pediatric obesity in China [6,7], research examining adherence to the 24-h movement guidelines may be timely. However, few studies have examined adherence to 24-h movement guidelines in China [7,13,20]. For example, Guan and colleagues [7] examined the proportion of children (aged 4.1 to 6.3 years) from three kindergartens in Beijing meeting movement guidelines suggested by the World Health Organization, and reported that just 15.0% met all three guidelines, and 2.7% did not meet any guidelines. The current research in this area of inquiry in China has focused on pre-school [7] or elementary-aged children [13,21], and no studies, to the knowledge of the authors, have examined proportions of adolescents meeting these health-related guidelines in China. Adolescence may be a particularly important age to examine, given that health-related behaviors become more volitional and less dictated by parents as children grow older [11,22] and the risk for pediatric obesity rises with age [1,23]. Thus, the primary purpose of this observational, cross-sectional analysis was to examine the proportions of adolescents in an eastern province in China who partially or fully meet the PA, screen-time, and sleep duration guidelines. 

Sociodemographic variables may influence the types of activities that youth choose, as well as barriers they experience when engaging in health-related behaviors. Gender, in particular, may be of particular relevance, given that several studies examining movement guideline adherence among children in China [22], as well as other contexts [12,19], have demonstrated that a lower proportion of girls than boys tend to meet these recommendations together and in isolation. This may be an important point in China, where evidence suggests that girls are at a higher risk of experiencing pediatric obesity than boys [10,24]. As such, a secondary purpose of this analysis was to examine whether gender differences emerged in the proportion of boys and girls meeting these 24-h movement guidelines. 

## 2. Methods

### 2.1. Study Design and Sample

This observational study collected data from a convenience sample of adolescents in a large regional high school in an eastern province of China. The total high school-aged adolescent population in the school was approximately 4500. Data were collected through self-reported survey in the fall semester (November–December) of 2019. The response rate was 35.9%, and 1618 students consented and participated in the data collection. The corresponding author’s institutional review board (Human Subject Research Ethics Committee) and the local school approved the study protocols. Parental consent was sought for those who were under 18 years of age, and participatory assent was obtained for all participants prior to data collection. 

### 2.2. Measures and Procedures

#### 2.2.1. Demographics

Adolescents’ age, ethnicity, gender, and parents’ highest education were collected in the self-reported survey. Specifically, adolescent age was collected as chronological age. Ethnicity included options of Han and minorities, gender included options of male and female, and parent education included options of (a) high school or less, (b) some college or bachelor’s degree, and (c) graduate or professional degree. 

#### 2.2.2. 24-h Movement Behaviors

Three questions were utilized to evaluate if participants met the 24-h movement guidelines. These items were modelled after the National Survey of Children’s Health from the United States Census Bureau (2019). To assess PA, adolescents were asked: “During the past week, on how many days did you exercise, play a sport, or participate in PA (that resulted in elevated heart rate, accelerated breathing, and/or sweating) for at least 60 min?” Eight response options were available ranging from “0” to “7 days.” A dichotomous variable, aligned with Tremblay and colleagues recommendations [11], was created where responses of “7 days” were coded as “meeting PA guidelines,” and all other responses were coded as “not meeting PA guidelines.” For screen-time, adolescents were asked: “On an average weekday, besides doing homework, about how many hours do you usually spend watching TV, online videos, browsing internet, or playing video games?” A dichotomous variable was then created where responses of “2 h or less” were categorized as meeting 24-h movement guidelines, and “more than 2 h” as not meeting 24-h movement guidelines. To assess sleep, an open-ended question asked: “During the past week, how many hours of sleep did you have on an average weeknight?” Responses within the range of 8–10 h for 14–17 years olds [11], and 7–9 h for 18–19 year olds [25] were coded as “meeting sleep guidelines,” and all other responses were coded as “not meeting sleep guidelines.” 

### 2.3. Data Analysis

During the data screening, participants with missing or invalid question responses (*n* = 280) were listwise removed. As such, the final sample subjects included in the data analysis were only the ones with complete responses (*N* = 1338). We analyzed the data in two steps: A frequency analysis was conducted to summarize the participants demographics and the number of 24-h movement guidelines met of the total sample and by gender. The bias-corrected and accelerated bootstrap 95% confidence interval (CI) was estimated based on 1000 bootstrap sample [26]. Next, for cells with sufficient sample counts (*n* > 5), we used chi-squared tests to compare the differences in the proportion of meeting different 24-h movement guidelines, independently and jointly, between female and male participants [27]. The data analyses were conducted using SPSS (Ver. 25, IBM; Armonk, NY, USA). Statistical significance was held at the 0.05 level. 

## 3. Results

**Figure 1:**
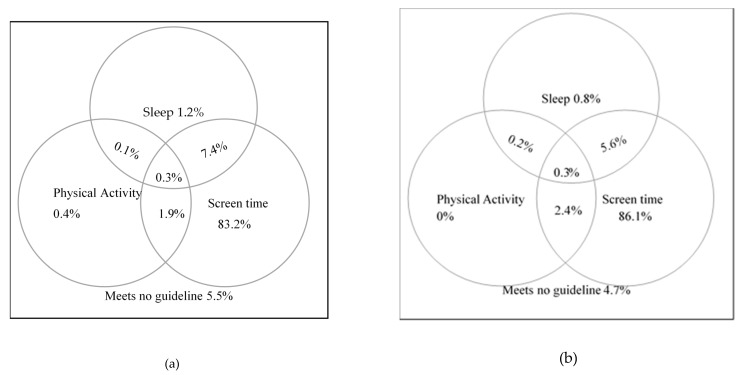
Percentages meeting 24-h movement guidelines among male (**a**) and female (**b**) adolescents.

The sample was gender-balanced, consisting of 49.4% (95% CI = 46.9%–52.0%) females, but included predominantly Han ethnics 97.5% (95% CI = 96.7%–98.3%), which was consistent with the local population. On average, the participants were 16.67 years old (SD = 0.68), ranging from 14 to 19 years old. As seen in Table 1, about half of the participants had parents with some college (47.8%) or graduate degrees (7.0%). A high proportion of adolescents did not meet the PA (97.2%, 95% CI = 96.2%–98.0%) or age-appropriate sleep (92.1%, 95% CI = 90.6%–93.5%) guidelines, but met the screen-time viewing (93.6%, 95% CI = 92.4%–94.7%) guideline. Overall, only 0.3% (95% CI = 0.1%–0.6%) of the sample met all three of the 24-h movement guidelines, 8.8% (95% CI = 7.5%–10.2%) met two, 85.8%% (95% CI = 84.0%–87.4%) met one, and 5.1% (95% CI = 4.0%–6.4%) met none (Table 1 and Figure 1).

There was no statistically significant percentage difference between female and male participants in meeting PA (Δ% = 0.1%, *χ*^2^ = 0.01, *p* = 0.91), screen-time viewing (Δ% = 1.6%, *χ*^2^ = 1.43, *p* = 0.23), or age-appropriate sleep hour guidelines (Δ% = 2.2%, *χ*^2^ = 2.20, *p* = 0.14), independently. As shown in Figure 1, when examining the combination of these guidelines met, for those cells with sufficient subject counts (*n* > 5), we did not find statistically significant percentage differences between female and male participants in meeting sleep and screen-time viewing guidelines jointly (Δ% = 1.8%, *χ*^2^ = 1.78, *p* = 0.18), or screen-time viewing and PA jointly (Δ% = 0.5%, *χ*^2^ = 0.40, *p* = 0.53). Similarly, there was no significant difference between female and male participants in meeting 0, 1, or 2 guidelines as shown in Table 1 (all *p* values > 0.05). 

## 4. Discussion 

The unique contribution of this study is the analysis of the proportions of adolescents in China who individually and jointly adhered to the 24-h movement guidelines. Prior research supports the assertion that the whole day matters [11], where the synergistic effects of adherence to all three 24-h movement behavior guidelines outweighs the benefits of meeting guidelines in isolation [11,13,14,15,16,17,18]. This includes findings focusing on pediatric obesity, where adolescents who meet all three guidelines are at a lower risk of experiencing pediatric obesity than those who meet zero, one, or two [13,14,15,16,17,18]. Of concern, few adolescents (0.3%) adhered to all three 24-h movement guidelines in this study. This figure appears much lower than many estimates in prior research internationally, which tend to range from 7.2% [13] to 18.4% [18]. As such, most adolescents in this study (99.7%) did not meet all three 24-h movement guidelines and therefore may not receive the collective, integrative physiological and psychological benefits associated with meeting these guidelines [5,11,13,14,15,16,17,18]. 

Of the three 24-h movement guidelines, it is clear that considerations to improve adherence to two of them, PA and sleep, are critical. Alarmingly, just 2.8% and 7.9% of the participants met the PA and sleep guidelines, respectively. Given the high percentage of adolescents meeting the screen-time viewing guideline (93.6%), these two guidelines are gateways for adolescents to enjoy the integrative benefits of meeting all three 24-h movement guidelines. It is of little surprise that adherence to PA guidelines was the lowest among the three health-related guidelines, given that this is a common trend in research among children in China [13] as well as adolescents abroad [12]. One potential explanation for why adherence to PA recommendations may be low in China is related to academic burden or stress. Academic burden or stress has been identified as a factor that plays a role in reducing the likelihood of adolescents meeting PA guidelines in countries with rigorous academic standards, like China [28,29]. For example, in a cross-sectional analysis of 48,118 school-aged children in Shanghai [29], academic burden was cited as the primary reason for youth not engaging in enough PA, and those who did not report academic burden were about five times more likely to meet PA recommendations. Specifically, high school adolescents on average spent more than 2 h per day on academic homework [29], although the amount of homework time that was also screen time is not clear. As such, assisting youth in finding a balance between time spent engaged in schoolwork (e.g., homework, studying) and PA time may be one strategy for enhancing PA recommendation adherence among Chinese adolescents. 

From a 24-h movement behavior perspective, it could be argued that meeting PA guidelines is linked to meeting sleep guidelines. For example, recent cross-sectional data have demonstrated that youth who regularly meet sleep duration guidelines are also more physically active and have lower rates of pediatric obesity [30,31]. Thus, the benefits associated with meeting sleep guidelines extend beyond simply influencing health-related outcomes, but also can contribute to adherence to other movement behaviors. The low adherence to the sleep guideline in this study was surprising, given adherence to meeting sleep guidelines tends to exceed 40% in studies internationally [12,13,15,16]. The high academic burden during high school years, which has been shown to impact PA participation and screen-time viewing among youth in China [29], might be related to their sleep duration as well.

Recommendations to enhance adherence to the sleep guideline, which would also be relevant for each of the 24-h movement guidelines, may adopt the 5 A’s Behavior Change Model recommended by Dosh and colleagues [32]. The 5 A’s Behavior Change Model includes asking potentially interested youth about the behaviors, advising youth and parents on the importance of the behaviors, assessing potential barriers, assisting with overcoming those barriers with best practices, and arranging for follow-up assessments, if needed. Whereas this model has been used in a variety of contexts to enhance adherence to healthy behaviors [32,33], we are unaware of its adoption, thus far, to enhance adherence to 24-h movement guidelines in totality. 

Examining the influence of gender on adherence to the 24-h movement guidelines can help identify if one group needs specialized, focused interventions to enhance adherence. In this study, no significant difference was found between the percentages of girls and boys with regard to meeting each of the guidelines singularly or concurrently. This finding is somewhat surprising, as it conflicts with research examining young children in China [7,24], as well as adolescents in other contexts [14], where boys are more likely to meet 24-h movement guidelines. It appears that, perhaps, factors influencing adherence to the 24-h movement guidelines among adolescents in this particular high school context (e.g., academic burden) may influence movement behaviors among boys and girls in similar ways. Therefore, these findings suggest that the need for intervention to enhance PA and sleep duration guideline adherence is not gender dependent, and therefore efforts to reduce barriers to health-related behaviors should be provided for both groups. 

This analysis has several strengths, including the use of a large sample of adolescents in an understudied geographic area and the adoption of a current movement framework to understand health behaviors. However, limitations are also evident. The primary limitation of this study may be the utilization of self-report measures, rather than objective measures, to measure PA, screen-time, and sleep. While self-report instruments have historically acted as a main source of information on how individual’s spend time, recent suggestions to move away from self-report measures cite limitations with the level of detail needed to examine movement across the 24-h period, as well as concerns related to the influence that social desirability can have on responses where participants may over-report desirable behaviors such as physical activity [34,35]. However, the utilization of self-report may have been advantageous to gain access to a group of individuals in an economically efficient manner that would be challenging otherwise. In addition, just one question was used for each 24-h movement behavior, making it challenging to evaluate the reliability of responses, and the way in which responses were dichotomized may limit variability and represent a narrow viewpoint on what PA, screen-time, and sleep participation are. Finally, while the sample size is relatively large for this study, it is nevertheless a convenient sample, representing an area of an eastern province in China. As such, interpretations of the study findings may be limited to the specific geographic region. 

## 5. Conclusions

In conclusion, while over 90% of the adolescents in this study met the screen-time viewing guideline of 2 h or less, over 90% of them did not meet the PA guideline of 60 min per day, or age-appropriate hours of sleep (8–10 h for 14–17 year olds, 7–9 h for 18–19 years olds). Consequently, less than 1% of the participants met all three, less than 10% met two, and about 85% met one of the 24-h movement guidelines. About 5% of the participants met none of the guidelines. Additionally, contrary to some existing reports, we did not find statistically significant difference in the percentage of adolescents meeting these guidelines in their singular or joint status, between female and male participants. 

## Figures and Tables

**Table 1:** Demographic characteristics of adolescents in high school.

Age Range	14–19 Years Old	Female	Male
	*n* = 1338	*n* = 661	*n* = 677
Age (y), mean (SD)	16.67 (0.68)	16.62 (0.69)	16.71 (0.68)
Gender (%, 95% CI)			
Female	49.4% (46.9–52.0%)	—	—
Male	50.6% (48.1–53.0%)	—	—
Ethnicity (%, 95% CI)			
Han	97.5% (96.7–98.3%)	97.0% (95.8–98.0%)	98.1% (97.2–99.0%)
Minority	2.5% (1.8–3.1%)	3.0% (2.0–4.2%)	1.9% (1.2–2.7%)
Parent Education (%, 95% CI)			
High school or less	45.2% (42.8–47.6%)	42.8% (39.0–46.6%)	47.6% (43.9–51.3%)
Some college or bachelor’s degree	47.8% (45.3–50.3%)	50.5% (46.8–54.3%)	45.1% (41.5–48.6%)
Graduate or professional degree	7.0% (5.6–8.4%)	6.7% (4.8–8.6%)	7.4% (5.8–9.2%)
PA participation (%, 95% CI)			
Less than 60 min/day	97.2% (96.2–98.0%)	97.1% (95.9–98.2%)	97.2% (96.0–98.2%)
60 min or more/day	2.8% (2.2–3.6%)	2.9% (2.0–3.9%)	2.8% (1.8–4.0%)
Average weeknight sleep (%, 95% CI)			
Below age-appropriate hours	92.1% (90.6–93.5%)	93.2% (91.5–94.9%)	91.0% (88.8–93.1%)
Met age-appropriate hours	7.9% (6.7–9.3%)	6.8% (5.0–8.8%)	9.0% (7.1–10.9%)
Screen-time viewing (%, 95% CI)			
2 h or less/day	93.6% (92.4–94.7%)	94.4% (92.7–96.1%)	92.8% (90.7–94.8%)
More than 2 h/day	6.4% (5.2–7.7%)	5.6% (4.1–7.1%)	7.2% (5.5–8.9%)
24-h movement guideline (%, 95% CI)			
Met 0 guideline	5.1% (4.0–6.4%)	4.7% (3.3–6.2%)	5.5% (4.0–6.9%)
Met 1 guideline	85.8% (84.0–87.4%)	86.8% (84.6–89.0%)	84.8% (82.0–87.3%)
Met 2 guidelines	8.8% (7.5–10.2%)	8.2% (6.4–10.0%)	9.5% (7.4–11.7%)
Met 3 guidelines	0.3% (0.1–0.6%)	0.3% (0.0–0.8%)	0.3% (0.0–0.7%)

CI: confidence interval; SD: Standard deviation.
